# Adenosine A_2A_ Receptor Gene Knockout Prevents l-3,4-Dihydroxyphenylalanine-Induced Dyskinesia by Downregulation of Striatal GAD67 in 6-OHDA-Lesioned Parkinson’s Mice

**DOI:** 10.3389/fneur.2017.00088

**Published:** 2017-03-21

**Authors:** Su-bing Yin, Xiao-guang Zhang, Shuang Chen, Wen-ting Yang, Xia-wei Zheng, Guo-qing Zheng

**Affiliations:** ^1^Department of Neurology, The Second Affiliated Hospital and Yuying Children’s Hospital of Wenzhou Medical University, Wenzhou, China

**Keywords:** Parkinson’s disease, dyskinesia, adenosine A_2A_ receptor, striatum, glutamic acid decarboxylase

## Abstract

l-3,4-Dihydroxyphenylalanine (l-DOPA) remains the primary pharmacological agent for the symptomatic treatment of Parkinson’s disease (PD). However, the development of l-DOPA-induced dyskinesia (LID) limits the long-term use of l-DOPA for PD patients. Some data have reported that adenosine A_2A_ receptor (A_2A_R) antagonists prevented LID in animal model of PD. However, the mechanism in which adenosine A_2A_R blockade alleviates the symptoms of LID has not been fully clarified. Here, we determined to knock out (KO) the gene of A_2A_R and explored the possible underlying mechanisms implicated in development of LID in a mouse model of PD. A_2A_R gene KO mice were unilaterally injected into the striatum with 6-hydroxydopamine (6-OHDA) in order to damage dopamine neurons on one side of the brain. 6-OHDA-lesioned mice were then injected once daily for 21 days with l-DOPA. Abnormal involuntary movements (AIMs) were evaluated on days 3, 8, 13, and 18 after l-DOPA administration, and real-time polymerase chain reaction and immunohistochemistry for glutamic acid decarboxylase (GAD) 65 and GAD67 were performed. We found that A_2A_R gene KO was effective in reducing AIM scores and accompanied with decrease of striatal GAD67, rather than GAD65. These results demonstrated that the possible mechanism involved in alleviation of AIM symptoms by A_2A_R gene KO might be through reducing the expression of striatal GAD67.

## Introduction

Parkinson’s disease (PD) is a common progressive neurodegenerative disorder characterized by bradykinesia, rigidity, resting tremor, and postural instability (1). The pathogenesis of PD has not been completely delineated, but believed to involve a multifactorial etiology including age-related, genetic, and environmental factors (2). Long-term therapy with l-3,4-dihydroxyphenylalanine (l-DOPA), the most common and effective symptomatic treatment for PD, often leads to the severe side effects such as dystonic, choreic, and ballistic movements, collectively referred to as l-DOPA-induced dyskinesia (LID) (3). Approximately 10% of patients have experienced LID in the first year of dopamine replacement therapy, and the incidence of LID is up to nearly 95% in patients who survive 15 years from diagnosis (4, 5). LID severely affects quality of life and leads to enormous social and economic burden to patients and their caregivers. Current effective treatment strategy for LID is mainly by a reduction in drug dose, which has a rebound of parkinsonian symptoms (6). Thus, an effective and satisfying management of LID is still urgently needed of PD therapy.

Basal ganglia is an important subcortical center for the regulation of voluntary movement, whose output pathways include the direct striatonigral–striatoentopeduncular gamma aminobutyric acid (GABA) pathway and the indirect striatopallidal GABA pathway (7). The unbalanced activities of basal ganglia output pathways were thought to be correlated with the development of the dyskinesias. Especially dysfunction of the indirect pathway can indirectly cause alterations in the pallidothalamic output (8). Adenosine A_2A_ receptor (A_2A_R), which acts in opposition to dopamine D2 receptor (D2R) and colocalized with D2R in indirect striatopallidal GABAergic pathway, offers a unique opportunity to modulate basal ganglia function mediated by dopamine (9). Pharmacological antagonists of A_2A_R prevented dopamine-induced motor complications and significantly increased l-DOPA efficacy in rats and primates when administered with l-DOPA (10, 11). On the basis of recent preclinical studies performed using A_2A_R antagonists as well as the good curative effect obtained, A_2A_R antagonists appear to be a promising non-dopaminergic therapy for PD (12–14). Unfortunately, the mechanism by which adenosine A_2A_R blockade alleviates the symptoms of LID has not been fully clarified.

As the neurotransmitter of striatal projection neurons, GABA might play a role in LID (15). Studies have shown that the chronic systemic administration of l-DOPA to 6-OHDA-lesioned rats increased GABA release in the substantia nigra (16, 17) and GABA-synthesizing enzyme glutamic acid decarboxylase (GAD) gene expression in striatonigral neurons (18–20). GAD, the rate-limiting enzyme responsible for the production of GABA, is classified into two major types: GAD65 and GAD67. GAD65 and GAD67 are derived from two different genes, which are located on chromosome 10 and chromosome 2, respectively (21). The reason why GAD67 and GAD65 are so named is that their apparent molecular weights are 65,400 and 66,600 Da, respectively. GAD65 is largely present as an inactive apoenzyme (GAD without bound cofactor; apoGAD), thus providing a reservoir of inactive GAD that can be drawn on when additional GABA synthesis is required, while GAD67 is by and large present as a permanently active pyridoxal phosphate-bound holoenzyme (22–24). A regional difference exists in the relative expression of these two isoforms of GAD. Comparative *in situ* hybridization in rat brain showed high relative levels of GAD67 mRNA were expressed in brain regions such as medial septal nucleus, neocortex, inferior colliculus, and cerebellum, while GAD65 mRNA was particularly abundant in regions such as olfactory tubercle, zona incerta, and substantia nigra (25). In the matter of subcellular distributions, GAD65 was preferentially found in nerve terminals, while GAD67 was located more equally in the cytoplasm as well as in nerve terminals, which suggested that GAD67 might be involved in the synthesis of GABA for general metabolic activity, whereas GAD65 was involved in synaptic transmission (26). Several lines of evidence have revealed that LID in experimental models of PD was associated with an overexpression of GAD mRNA on striatal output neurons (19, 20, 27–29). Between these two neurotransmitter-related mRNAs, GAD67 mRNA has mainly been the focus of the study and considered to be positively correlated with the dyskinesia. The upregulation of GAD67 mRNA following the dopamine-denervated lesion was paralleled by increased GAD enzyme activity and extracellular levels of GABA in the striatum (28, 30). In addition, the antagonistic adenosine A_2A_ and D2R interaction occurring in striatopallidal GABAergic pathways also regulates GABAergic neurotransmission in these areas (31). When adenosine levels rise in the basal ganglia, the activation of A_2A_R abolished dopamine D2 agonist-induced inhibition of GABA release (7, 32–34). Whereas A_2A_R antagonists could potentiate this inhibitory effect and reduced the release of GABA (31).

Based on these findings, it can be hypothesized that there is an association between adenosine A_2A_R and GAD. Thus, the aim of our study is to explore the effect of adenosine A_2A_R knockout (KO) on the expression of GAD67 and GAD65 in striatal neurons in LID.

## Animals and Methods

### Animals

The A_2A_R KO mice were purchased from the Jackson Laboratory and were established on the Balb/c strain background. Wild-type (WT) Balb/c mice were purchased from Slac Laboratory Animal Company. KO mice were crossed with WT mice to get the heterozygous mice, and then, the heterozygous mice were crossed with each other to get the KO mice and WT mice. A_2A_R homozygous KO mice and WT Balb/c mice were bred at the Laboratory Animal Centre of Wenzhou Medical University. Experiments were conducted on 24 WT and 24 adenosine A_2A_R gene KO adult male mice weighing 25–30 g on arrival, which was further randomly assigned to the following three experimental groups (eight per group): the sham group, the PD group, and the LID group. All of the mice were housed under a 12-h light:12-h dark cycle, temperature 22.0 ± 2.0°C, and relative humidity of 55 ± 10%. Food and water were available *ad libitum*. All procedures associated with the care of animals were performed according to the National Institutes of Health for the care and use of laboratory animals (NIH publication No 80-23) and were approved by the Animal Ethics Committee of Wenzhou Medical University. All efforts were made to reduce the number of animals used.

### Induction of Parkinsonism and LID

Unilateral 6-OHDA lesions were performed according to the previous standard procedure (35, 36). Briefly, mice were anesthetized with chloral hydrate (0.3 g/kg, i.p.) and placed in the stereotaxic frame. Two microliters of 6-OHDA (1 mg 6-OHDA dissolved in 200 μl 0.05% ascorbic acid) were stereotaxically injected into the left striatum (relative to bregma and dura): anterior–posterior: −0.5 mm, medial–lateral: +2.0 mm, and dorsoventral: −2.8 mm with a Hamilton syringe at a speed of 0.4 μl/min. The sham-operated mice were similarly treated but received physiological saline containing 0.2% ascorbic acid instead of 6-OHDA. After injections, mice were kept warm (37°C) until they recovered from surgery. Three weeks later, the lesioned mice were evaluated by rotation test over 30 min after apomorphine (0.5 mg/kg, i.p.) administration, and only severely lesioned animals were selected for the next experiment (more than 7 full-body turns/min toward the side of the unlesioned side). One day after apomorphine test, they were then treated with 25 mg/kg l-DOPA and 6.25 mg/kg benserazide (i.p. injection/day) for 3 weeks in order to induce a mouse model of dyskinesia.

### Abnormal Involuntary Movement (AIM) Ratings

Abnormal involuntary movements were evaluated on days 3, 8, 13, and 18 after l-DOPA administration. After the daily injection of l-DOPA, the mice were observed individually for 1 min every 20 min from 20 to 120 min. At each observation time point, the AIMs were classified into three subtypes: axial, limb, and orolingual movements as detailed by Lindenbach et al. (37). Each subtype was scored on a severity scale from 0 to 4 (0 = absent, 1 = present during less than half of the observation time, 2 = present for more than half of the observation time, 3 = present for 1 min but suppressible by external stimuli, and 4 = present all the time but not suppressible by external stimuli). For each mouse, the total AIM score for each test session was calculated by summing the three individual dyskinesia scores.

### Real-time Polymerase Chain Reaction

The expression levels of GAD65 and GAD67 were measured using real-time polymerase chain reaction. Total RNA was extracted from the brain striatum of lesioned hemisphere using TriZol (Ambion, USA) and reverse transcribed into cDNA using the PrimeScript RT Reagent Kit (Takara, Japan). Quantitative polymerase chain reaction was performed in a 25-μl reaction mixture using SYBR Premix Ex Taq™ II (Takara, Japan) according to the manufacturer’s instructions. The amplification of the housekeeping gene β-GAPDH cDNA was used as an internal control. Forward and reverse primers, respectively, used for PCR were 5′-CACCTGCGACCAAAAACCC-3′ and 5′-AGATGACCATGCGGAAGAAG-3′ for GAD65, and 5′-CTCCCTTCTTCAGGCTCTCCC-3′ and 5′-GGTCTTGGGGTCTCTACGGTTC-3′ for GAD67.

### Histological and Immunohistochemical Analyses

In terms of histological and immunohistochemical analysis, we extract the whole striatum section (both dorsal and ventral) to stain. Experimental mice were anesthetized with 10% chloral hydrate (0.3 g/kg, i.p.), and perfused transcardially with saline and then fixed by perfusion with 4% paraformaldehyde. Brains were removed and prepared as 4-μm paraffin-embedded sections. Endogenous peroxidase was blocked with 3% H_2_O_2_, and tissue was treated overnight at 4°C with primary antibodies:mouse monoclonal antibody against GAD67 (1:1,000 dilution, Millipore), or rabbit polyclonal antibody against GAD65 (1:1,000 dilution, Sigma). Sections were then incubated with the secondary antibody for 1 h at room temperature, followed by coloration with 3,3-diaminobenzidine (DAB, Sigma, Germany) and hematoxylin counterstaining. The sections were washed with 0.01 mol/l PBS between each step. We analyzed the average integral optical density (IOD) of cells positively stained with GAD65 or GAD67 and calculated the mean values and SDs. Three regions of interest were randomly chosen from each section for quantification by using Image-Pro Plus 6.0.

### Statistical Analysis

Measurement data were expressed as mean ± SD. Significance of the difference between two groups was analyzed using two-tailed Student’s *t*-test. Multiple groups were compared using one-way analysis of variance (ANOVA) and followed by LSD *post hoc* comparisons when appropriate. All statistical analyses were performed with SPSS 20.0 software (IBM, Armonk, NY, USA) and GraphPad Prism 6.05 (GraphPad, La Jolla, CA, USA). *P* < 0.05 was considered statistically significant.

## Results

### Effects of Adenosine A_2A_R Gene KO on the Behavior of Mice with LID

**Figure 1:**
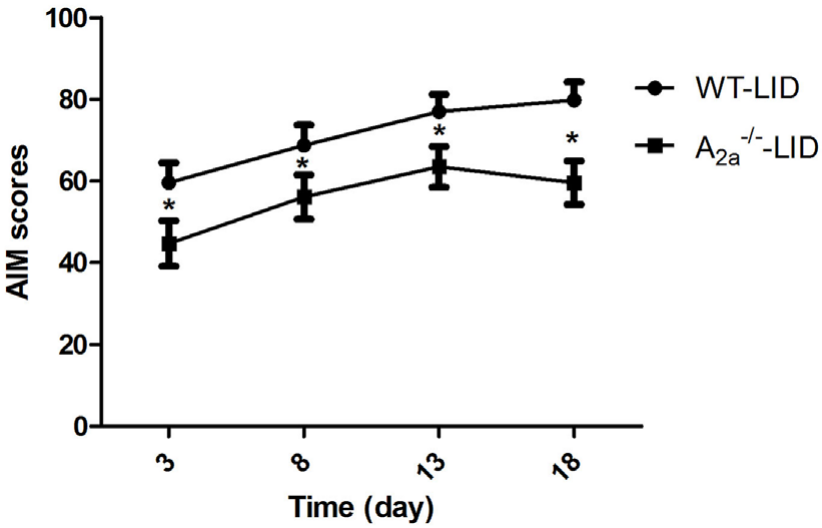
**Time course of abnormal involuntary movement (AIM) scores development with l-3,4-dihydroxyphenylalanine (l-DOPA) plus benserazide exposure**. AIM scores in wild-type (WT)-l-3,4-dihydroxyphenylalanine-induced dyskinesia (LID) group, A_2A_^−/−^ LID group on days 3, 8, 13, and 18 after l-DOPA administration. *P < 0.05, compared with WT-LID group.

In this study, in the process of induction of 6-OHDA-lesioned PD mice, the survival rate and the rate of successful lesions are about 80 and 50%, respectively. PD rats treated with l-DOPA for 18 days developed a progressive increase in LID as indicated by AIM score. Median AIM score of WT-LID increased from 59.63 ± 8.12 on day 3 to 79.88 ± 5.46 on day 18 (*n* = 8, *F*_time_ = 12.32, *P* < 0.05 compared with day 3, ANOVA and followed by LSD *post hoc* analysis, Figure 1). After KO of adenosine A_2A_R gene, similar trend of AIM score was found, namely, increased from 44.75 ± 7.61 on day 3 to 64.00 ± 7.80 on day 13, and 60.5 ± 6.5 on day 18 (*n* = 8, *F*_time_ = 0.523, *P* > 0.05 compared with day 13, ANOVA and followed by LSD *post hoc* analysis, Figure 1). It is remarkable that AIM score of A_2A_^−/−^ LID group was obviously lower compared to the WT-LID group at the same time point (*n* = 8, *P* < 0.05, two-tailed Student’s *t*-test, Figure 1).

### Effects of Adenosine A_2A_R Gene KO on GAD65 and GAD67 Expression in the Striatum of Mice with LID

#### Immunohistochemistry

**Figure 2:**
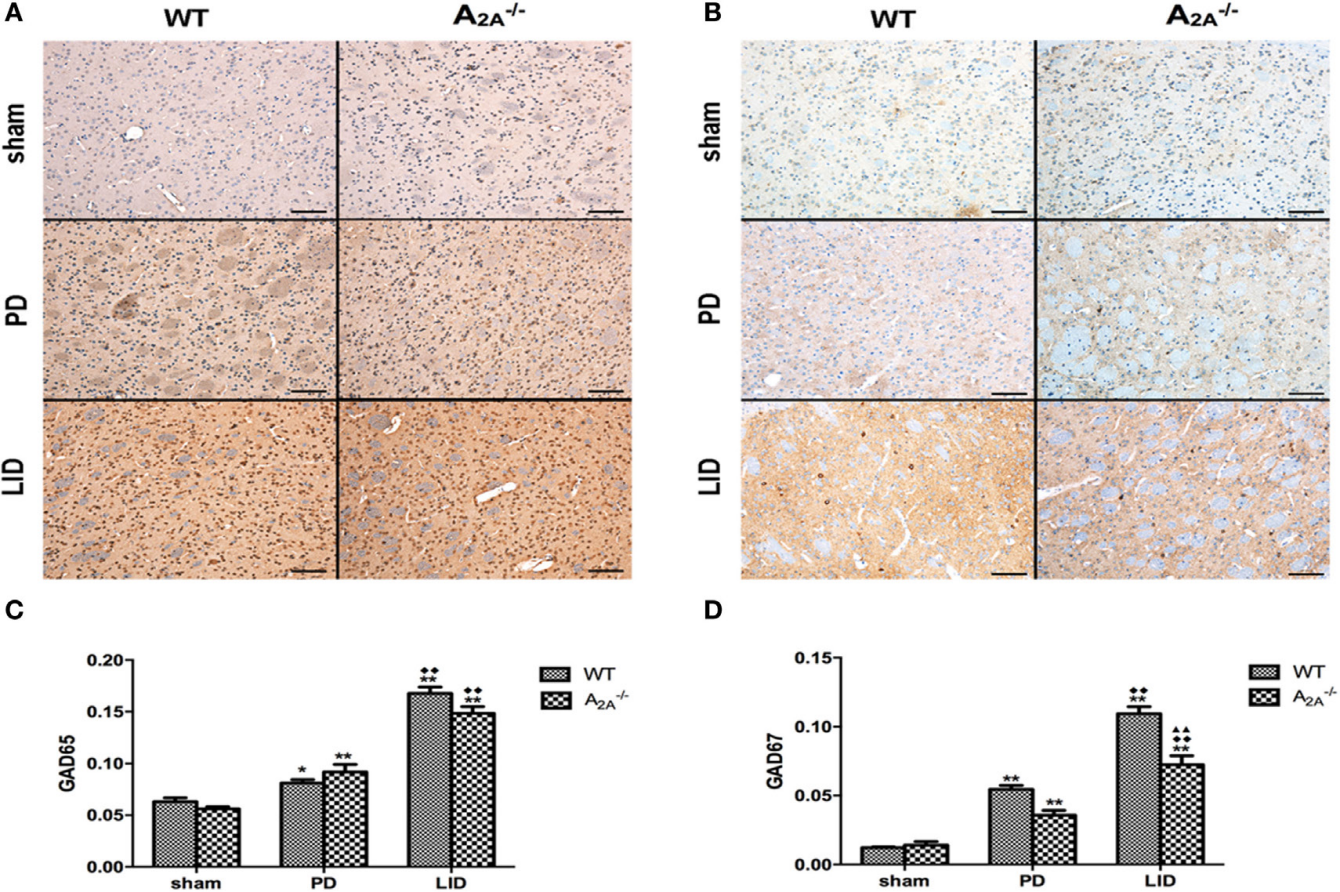
**Effects of adenosine A_2A_ receptor (A_2A_R) gene knockout (KO) on striatal GAD65 and GAD67 expression**. **(A)** Representative images of GAD65 immunohistochemistry studies in sham, 6-OHDA-lesioned wild-type (WT), or A_2A_R gene KO mice treated with vehicle or pulsatile l-3,4-dihydroxyphenylalanine (l-DOPA) (25 mg/kg, bid: 9:00 a.m.; 5:00 p.m.). **(B)** Representative images of GAD67 immunohistochemistry studies in sham, 6-OHDA-lesioned WT, or A_2A_R gene KO mice treated with vehicle or pulsatile l-DOPA (25 mg/kg, bid: 9:00 a.m.; 5:00 p.m.). **(C)** Statistical analysis for integral optical density (IOD) of GAD65. There was no significant difference of GAD65 between WT-l-3,4-dihydroxyphenylalanine-induced dyskinesia (LID) and A_2A_^−/−^ LID groups, *n* = 8 per group. *P < 0.05, **P < 0.01 vs. sham. ^♦♦^*P* < 0.01 vs. Parkinson’s disease (PD). **(D)** Statistical analysis for IOD of GAD67. The IOD of GAD67 in WT-LID group was significantly higher than A_2A_^−/−^ LID group, *n* = 8 per group. **P < 0.01 vs. sham. ^♦♦^*P* < 0.01 vs. PD. ^▲▲^*P* < 0.01 vs. LID.

Immunohistochemical studies showed significant increase of GAD65 (sham: 0.063 ± 0.007; PD: 0.081 ± 0.007; LID: 0.168 ± 0.013) and GAD67 (sham: 0.012 ± 0.001; PD: 0.055 ± 0.006; LID: 0.109 ± 0.010) in WT-LID compared to WT-sham and WT-PD groups (*n* = 4, *P* < 0.05, two-tailed Student’s *t*-test, Figure 2). In A_2A_R gene KO groups, treatment with l-DOPA (LID group) can significantly increase the IOD of both GAD65 (sham: 0.056 ± 0.004; PD: 0.092 ± 0.015; LID: 0.149 ± 0.013) and GAD67 (sham: 0.014 ± 0.005; PD: 0.036 ± 0.007; LID: 0.072 ± 0.013) (*n* = 4, *F*_treatment_ = 5.324, *P* < 0.05 vs. PD group or sham group, ANOVA and followed by LSD *post hoc* analysis, Figure 2). When comparing the IOD of GAD67, WT-LID group was significantly higher than A_2A_^−/−^ LID group (*n* = 4, *P* < 0.01, two-tailed Student’s *t*-test, Figure 2). However, there was no significant difference of GAD65 between WT-LID and A_2A_^−/−^ LID groups (*n* = 4, *P* > 0.05, two-tailed Student’s *t*-test, Figure 2).

#### Real-time Polymerase Chain Reaction

**Figure 3:**
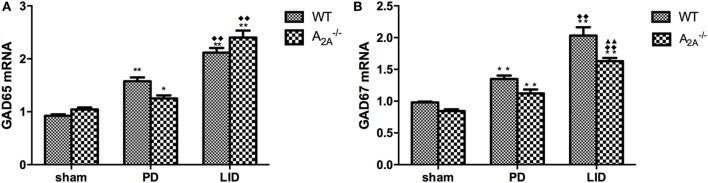
**Effects of adenosine A_2A_ receptor gene knockout on striatal GAD65 and GAD67 mRNA expression**. **(A)** There was no significant difference of striatal GAD65 mRNA expression between wild-type (WT)-l-3,4-dihydroxyphenylalanine-induced dyskinesia (LID) and A_2A_^−/−^ LID groups, *n* = 8 per group. *P* > 0.05 compared with A_2A_^−/−^ LID group. **(B)** In 6-OHDA-lesioned mice treated with l-3,4-dihydroxyphenylalanine (l-DOPA), striatal GAD67 mRNA level was significant lower in the A_2A_^−/−^ LID group compared to WT-LID group, *n* = 8 per group. **P < 0.01 compared with A_2A_^−/−^ LID group.

Real-time polymerase chain reaction showed significant increase of GAD65 (sham: 0.93 ± 0.05; PD: 1.58 ± 0.15; LID: 2.12 ± 0.18) and GAD67 (sham: 0.98 ± 0.03; PD: 1.35 ± 0.10; LID: 2.03 ± 0.26) mRNA expression in WT-LID compared to WT-sham and WT-PD groups (*n* = 4, *P* < 0.05, two-tailed Student’s *t*-test, Figure 3). In A_2A_R gene KO groups, treatment with l-DOPA (LID group) can significantly increase the mRNA expression of both GAD65 (sham: 1.05 ± 0.07; PD: 1.25 ± 0.11; LID: 1.55 ± 0.57) and GAD67 (sham: 0.85 ± 0.05; PD: 1.12 ± 0.12; LID: 1.63 ± 0.10) (*n* = 4, *F*_treatment_ = 11.23, *P* < 0.05 vs. PD group or sham group, ANOVA and followed by LSD *post hoc* analysis, Figure 2). In 6-OHDA lesioned mice treated with l-DOPA, striatal GAD67 mRNA level was significant lower in the A_2A_^−/−^ LID group compared to WT-LID group (*n* = 4, *P* < 0.01, two-tailed Student’s *t*-test, Figure 3). However, when comparing the mRNA expression of GAD65, the WT-PD group shows a milder increase compared to A_2A_^−/−^ PD group and a decrease in A_2A_^−/−^ LID group in contrast to WT-LID. Nevertheless, there was no significant difference between two groups (*n* = 4, *P* > 0.05, two-tailed Student’s *t*-test, Figure 3).

## Discussion

The present study showed that A_2A_R gene KO reduced the severity of LID induced by subchronic l-DOPA in 6-OHDA-lesioned mice. A_2A_R gene KO also reversed the effects of l-DOPA on GAD67, but not GAD65 expression in striatal projection neurons. These data supported the hypothesis that deletion of A_2A_R gene alleviated LID *via* downregulating the expression of striatal GAD67.

l-3,4-Dihydroxyphenylalanine remains the primary pharmacological agent for the symptomatic treatment of PD. However, the development of LID limits the long-term use of l-DOPA for PD patients. Recent studies showed that A_2A_R might play an important role in the treatment of LID. Thus, in the present study, we determined to KO the gene of A_2A_R and explored the possible underlying mechanisms implicated in development of LID in a mouse model of PD.

Previous experimental data have showed that chronic l-DOPA treatment significantly increased the level of striatal A_2A_R mRNA in normal monkeys exhibiting marked dyskinesia (38). Consistent with this, two recent positron emission tomography studies have reported that there were higher levels of A_2A_R binding in dyskinetic PD patients compared with non-dyskinesia (39, 40). Some data have reported that A_2A_R antagonists prevented dopamine-induced motor complications in 6-OHDA-lesioned rodent animal model of PD (41) and MPTP-treated primates (42). These findings provided overwhelming evidence for the assumption that blockade of A_2A_Rs on striatopallidal neurons of the indirect pathway may have a beneficial antidyskinetic potentiality (43).

As previous A_2A_R antagonists have been certified for their properties of inability to worsen established dyskinesia, we found that A_2A_R gene KO was effective in reducing AIM scores. The peak of AIM scores appeared beforehand at day 12 in A_2A_^−/−^ LID group, while WT-LID group was still in the process of developing LID. And severity of dyskinesia was significantly alleviated by A_2A_R KO during the whole observation period. These findings again provided directly strong evidence for the beneficial antidyskinetic potentiality of A_2A_R deficiency against the development of LID, which well verified the above assumption.

As described above, the A_2A_R gene-deficient mice induced to dyskinesia by administration of l-DOPA (25 mg/kg) presented a relatively low AIM score in our study. However, the mechanism of the alleviation of dyskinesia symptoms by A_2A_R KO is unclear. Possible pathway might include D2R, since A_2A_R is largely co-expressed with D2R and antagonizes D2R-mediated behavioral and neurochemical effects in the basal ganglia through a form of antagonistic A_2A_–D2 heteromeric receptor complexes in the striatum where it modulates dopaminergic neurons activity (44). Moreover, enhanced GABA release was found in dyskinetic rats (16), and studies have showed that A_2A_R could affect the release of GABA *via* D2R. For instance, blockade of striatal A_2A_R reduced the dopamine D2R-mediated GABA release, whereas activation of A_2A_R promoted the release of GABA (45). However, what should also be noted is that the regulation of striatopallidal neurons mediated by A_2A_R does not completely ascribe to antagonistic A_2A_R–D2R heterodimer since the stimulatory effect of adenosine A_2A_R antagonists on motor activity is still present in D2 KO mice (46, 47). Hence, endogenous adenosine *via* A_2A_R at least partly regulates the activity of striatopallidal transmission in a dopamine D2R-independent manner and that other mechanisms that can also affect GABA release in the striatum might be of importance upon dopamine depletion (13, 48).

Glutamic acid decarboxylase, as the rate-limiting enzyme responsible for the production of GABA, was reasonably taken into our consideration. Our finding that unilateral degeneration of nigrostriatal dopaminergic neurons increased striatal GAD65 and GAD67 gene expression was consistent with a previous study (49), which has been thought involved in the activation of the indirect striatopallidal GABA pathway (50). GABA neurons in the striatum appear as medium-sized spiny projection neurons, medium-sized aspiny interneurons, and large-sized aspiny interneurons, all of which have synaptic connections from dopaminergic nerve terminals. GAD-immunoreactive GABA neurons in the striatum constitute most of the medium-sized spiny neurons with efferent projections (51–53). Dopamine, as is well known, can inhibit downstream GABA release in the globus pallidus by binding to Gi-coupled D2Rs located in the striatum. The dopamine-denervated lesion increased extracellular levels of GABA and simultaneously upregulated GAD65 and GAD67 gene expression in the striatum. The increase in GAD67 gene expression is restricted to a small population (approximately 10% of all striatal GABA neurons) of medium-sized projection neurons (18, 54). Chronic intermittent administration of l-DOPA (20 mg/kg) to mice with 6-OHDA lesion induced dyskinesia, thereby leading to further increases in GAD65 and GAD67 gene expression. The progressive increase in GAD gene expression was paralleled by an increase in GABA synthesis in striatopallidal neurons (20). Thus, the massive increase of GAD induced by chronic intermittent l-DOPA (20 mg/kg) administration in the damaged striatum appeared to be related to dopamine neuron degeneration.

As found in the present study, A_2A_^−/−^ LID group was accompanied with a significant decrease in GAD67, which is involved in general metabolic processes activity and is considered an index of GABA neuron activity (55). However, it did not produce significant modifications in striatal GAD65. The effects of A_2A_R KO were more pronounced on GAD67 than on GAD65, suggesting that GAD65 might not play a similar role as GAD67 on the sensitized behavioral responses to l-DOPA (20). It seems that different mechanisms might be involved in the regulation of these two GAD genes in striatopallidal neurons (49). GABA is synthesized by two distinct enzymes GAD67 and GAD65 that differ in their cellular localization, functional properties, and cofactor requirements. GAD67 exists as both homo- and heterodimers in both soluble and membrane-bound forms, primarily within the cell soma but also to a limited degree in terminals (56). During the development, GAD67, but not GAD65, deleted homozygous mice die immediately after birth and had only less than 10% of normal brain GABA levels (57). In the adult, it is critical for maintaining intracellular GABA reserves for metabolic activity and other functions such as synaptogenesis and protection against neuronal injury, rather than for vesicular release. GAD67 protein contents can be influenced by the level of intraneuronal GABA, and this occurrence was not associated with a change in GAD67 mRNA levels (58, 59). Also, there appears to be isoform-specific physiological feedback, in that GAD67 mRNA and protein levels, rather than GAD65, were increased in cortical neurons after sensory learning (60, 61). This is the reason, at least in part, GAD67 contributed to alleviating LID of A_2A_R KO mice.

In summary, the present study indicated that A_2A_R KO had an important effect on the expression of striatal GAD67, which might affect the production of GABA and thus further alleviate dyskinesia.

## Author Contributions

G-qZ conceived and designed the experiment. S-bY, X-gZ, SC, W-tY, and X-wZ performed the experiments. S-bY analyzed and interpreted the data. X-gZ, S-bY, and G-qZ wrote the article. All the authors read and approved the final manuscript.

## Conflict of Interest Statement

The authors declare that the research was conducted in the absence of any commercial or financial relationships that could be construed as a potential conflict of interest.
